# Evaluation of liver regeneration and post-hepatectomy liver failure after hemihepatectomy in patients with hepatocellular carcinoma

**DOI:** 10.1042/BSR20190088

**Published:** 2019-08-23

**Authors:** Wen-Feng Gong, Jian-Hong Zhong, Zhan Lu, Qiu-Ming Zhang, Zhi-Yuan Zhang, Chang-Zhi Chen, Xu Liu, Liang Ma, Zhi-Ming Zhang, Bang-De Xiang, Le-Qun Li

**Affiliations:** 1 Hepatobiliary Surgery Department, Affiliated Tumor Hospital of Guangxi Medical University, Nanning, China; 2 Guangxi Liver Cancer Diagnosis and Treatment Engineering and Technology Research Center, Nanning, China; 3 General Medicine Department, The First People’s Hospital of Qinzhou, Qinzhou, China; 4 Hepatobiliary Surgery Department, The Fifth Affiliated Hospital of Guangxi Medical University, Guigang, China

**Keywords:** HCC, hepatectomy, liver function, liver regeneration, liver volume

## Abstract

**Aim:** To explore clinical factors associated with extent of liver regeneration after hemihepatectomy to treat hepatocellular carcinoma (HCC).

**Methods:** Future liver remnant volume (as a percentage of functional liver volume, %FLRV) and remnant liver volume were measured preoperatively and at 1, 5, 9, and 13 weeks postoperatively.

**Results:** After hepatectomy, 1 of 125 patients (0.8%) died within 3 months, 13 (10.4%) experienced liver failure, and 99 (79.2%) experienced complications. %FLRV was able to predict liver failure with an area under the receiver operating characteristic curve of 0.900, and a cut-off value of 42.7% showed sensitivity of 85.7% and specificity of 88.6%. Postoperative median growth ratio was 21.3% at 1 week, 30.9% at 5 weeks, 34.6% at 9 weeks, and 37.1% at 13 weeks. Multivariate analysis identified three predictors associated with liver regeneration: FLRV < 601 cm^3^, %FLRV, and liver cirrhosis. At postoperative weeks (POWs) 1 and 5, liver function indicators were significantly better among patients showing high extent of regeneration than among those showing low extent, but these differences disappeared by POW 9.

**Conclusions:** FLRV, %FLRV, and liver cirrhosis strongly influence extent of liver regeneration after hepatectomy. %FLRV values below 42.7% are associated with greater risk of post-hepatectomy liver failure.

## Introduction

Hepatocellular carcinoma (HCC) is the most frequent primary liver cancer worldwide, as well as the third leading cause of cancer mortality [1]. Liver surgery, the primary curative option for HCC patients, can remarkably improve overall survival [2], but it should be performed only when the remaining liver can provide sufficient function. Inadequate future liver remnant volume (FLRV) can lead to post-hepatectomy liver failure (PHLF), which is a major cause of morbidity and mortality [3].

Hepatectomy, like portal vein embolization, appears to induce liver regeneration through a hyperplastic reaction [4–9]. The course of regeneration and what clinical factors may influence it are poorly understood, in part since previous studies have focused largely on liver progenitor cells [10] and animal models [11]. Few studies have examined patients specifically for the purpose of clarifying when and how post-hepatectomy liver regeneration occurs.

The present study examined liver regeneration growth ratios and extent of regeneration at different times after hemihepatectomy in HCC patients. Logistic regression was performed to identify factors associated with liver regeneration, and a clinical prediction model was constructed and validated. We also evaluated associations between liver regeneration and postoperative liver function.

## Patients and methods

### Patients

Patients undergoing hemihepatectomy in the Department of Hepatobiliary Surgery at The Affiliated Tumor Hospital of Guangxi Medical University between September 2013 and December 2016 were considered for enrollment in this prospective study. The present study was approved by the Ethics Committee of the Affiliated Tumor Hospital of Guangxi Medical University, and it complied with the Declaration of Helsinki. All patients gave written informed consent before being enrolled.

Patients were included if they (a) underwent initial hemihepatectomy, (b) were positive for serum hepatitis B surface antigen (HBsAg) before surgery, (c) had Child-Pugh grade A liver function, (d) showed <10% retention of indocyanine green at 15 min preoperatively, and (e) had HCC confirmed by post-hepatectomy histopathology. Patients were excluded if (a) they experienced tumor recurrence or progression during follow-up; (b) received transarterial chemoembolization (TACE), chemotherapy, portal vein embolization, or other anti-HCC treatments prior to hemihepatectomy; (c) lacked follow-up computed tomography (CT) and/or clinical data; (d) received TACE, chemotherapy, or other anti-HCC treatments postoperatively; or (e) were lost to follow-up.

### Preoperative planning and measurement of liver characteristics

Hepatectomy was planned to use Myrian-XP-Liver software (Intrasense, Montpellier, France), which relies on multidetector CT to provide three-dimensional visualization and measurement of liver structures (Supplementary Figure S1A,B). This program was used preoperatively to estimate total liver volume, functional liver volume (FLV), FLRV, remnant liver volume, resected liver volume, and tumor volume as described [12]. The three-dimensional model was manually divided into the resected and remnant areas along the principal plane of the liver defined by the middle hepatic vein and the gallbladder fossa. The parameter %FLRV was calculated by dividing FLRV by the functional liver volume before hemihepatectomy.

The program was also used at postoperative weeks (POWs) 1, 5, 9, and 13 to calculate remnant liver volume. Supplementary Figure S1C–F shows CT images of the same layer of one patient at different time points.

### Hepatectomy and patient management

Hemihepatectomy was performed as described [13] with the patient under general anesthesia in the supine position. The left/right artery and portal vein were individually dissected, ligated, and divided. The liver tissue was surgically removed from the bottom of the gallbladder to the liver secondary portal along the line section of ischemia. The middle-hepatic vein was exposed and retained completely during resection. Pringle’s maneuver was employed when necessary. The left/right bile duct and left/right hepatic vein were ligated, divided, and closed after hepatic parenchymal resection. Intraoperative data were recorded on the type of hemihepatectomy, blood loss volume, duration of porta hepatis clamping, and duration of surgery. Data were also recorded on transfusion of red cells and blood plasma as well as infusion of human blood albumin during hepatectomy.

All patients were assayed preoperatively for HBsAg, HBV DNA, total bilirubin, prothrombin time, serum albumin, prealbumin, alpha-fetoprotein, platelet count, and indocyanine green retention at 15 min. The liver in all patients was imaged preoperatively using multidetector CT. After hemihepatectomy, a blood test was conducted to evaluate liver function and clotting ability. The liver was imaged by multidetector CT at POWs 1, 5, 9, and 13.

### Liver regeneration outcomes

Growth ratio was calculated at POWs 1, 5, 9, and 13 using the equation [14,15]:

$$\text{Growth }{\text{ratio}}_{\text{POWn}}=\left[\frac{\left(\text{Remnant liver }{\text{volume}}_{\text{POWn}}-\text{FLRV}\right)}{\text{FLRV}}\right]\times 100\%.$$

Net growth ratio was calculated using the equations:

$${\text{Net growth ratio}}_{\text{POW}1}=\left[\frac{\left({\text{Remnant liver volume}}_{\text{POW}1}-\text{FLRV}\right)}{\text{FLRV}}\right]\times 100\%,$$

$$\text{Net growth }{\text{ratio}}_{\text{POW}5/9/13}=\left[\frac{\left(\text{Remnant liver }{\text{volume}}_{\text{POW}5/9/13}-\text{Remnant liver }{\text{volume}}_{\text{POW}1/5/9}\right)}{\text{Remnant liver }{\text{volume}}_{\text{POW}1/5/9}}\right]\times 100\%.$$

### Other outcomes

Post-hepatectomy liver failure was defined using “50–50 criteria” [16] as total bilirubin >50 μmol/L and prothrombin time < 50% on postoperative day 5. Postoperative complications were classified according to the 2004 Dindo–Clavien scheme [17] as major (grades III–V) or minor (grades I–II). Liver cirrhosis was defined histologically as F4 using ISHAK classification [18]. For stratifying patients by age, the median age of 47 years in our study population was used as the cut-off for defining patients as younger or older. For stratifying patients by growth ratio, the median value of 21.3% at POW 1 was used as the cut-off for defining patients as showing low or high extent of liver regeneration.

### Data collection and statistical analysis

Demographic, clinical, pathological, and follow-up data were entered prospectively into a central hospital database. SPSS 19.0 (IBM, U.S.A.) was used for all statistical analyses, with the threshold of significance defined as a two-tailed *P*<0.05. Data for continuous variables were expressed as median (range), while data for categorical variables were expressed as number (percentage). Intergroup differences in continuous variables were assessed for significance using Student’s *t*-test (if data were normally distributed) or the Mann–Whitney *U*-test (if data were skewed). Intergroup differences in categorical data were assessed using the *χ*^2^-test (two-tailed). Binary logistic regression was used to perform uni- and multivariate prediction of high extent of liver regeneration. Predictive power was assessed using a receiver operating characteristic (ROC) curve. MedCalc 9.2.0.1 was used to determine the cut-off %FLRV value that predicted PHLF.

The multivariate modeling was processed in R (version 3.3.3, www.r-project) to construct a nomogram to predict liver regeneration, the performance of which was assessed using bootstrapping with 1000 repetitions following by calculation of the area under the receiver response curve and associated 95% confidence interval (95%CI).

## Results

### Patient characteristics

**Figure 1:**
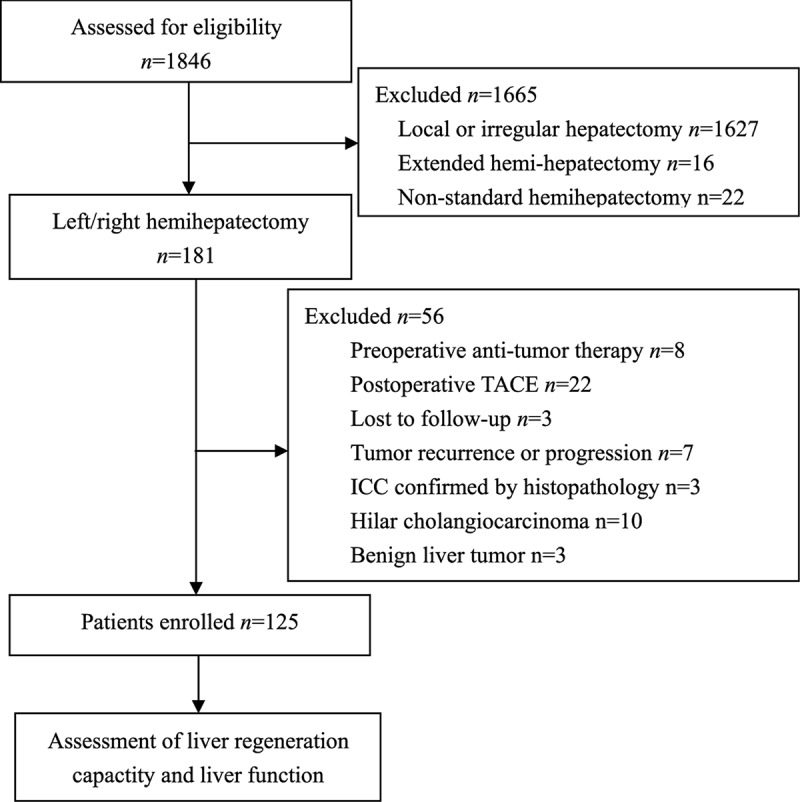
Flowchart of patient enrollment and assessment

Of the 1846 potentially eligible patients, 1721 were excluded and the remaining 125 were included, comprising 103 men and 22 women (Figure 1). Median body mass index was 21.5 kg/m^2^, body surface area was 1.61 m^2^ and median age was 47 years nb(range, 20–72). Just over half the patients (72, 57.6%) underwent right hemihepatectomy, and nearly two-thirds of all patients (77, 61.6%) had liver cirrhosis confirmed by postoperative histopathology. Pre- and perioperative patient characteristics are shown in Table 1.

**Table 1:** Clinical characteristics of patients

Characteristic	Value
Gender, M/F	103/22
Age, year	47 (20–72)
Body mass index, kg/m^2^	21.5 (15.4–31.3)
Body surface area, m^2^	1.61 (1.34–1.92)
Diabetes mellitus	7 (5.6%)
Preoperative MELD score	24 (12–29)
BCLC stage A/B/C	41/22/62
Type of hemihepatectomy	
Left	53 (42.4%)
Right	72 (57.6%)
Positive for hepatitis B virus DNA	80 (64%)
Previous antiviral therapy	51 (40.8%)
AFP, ≥200 μg/ml	70 (56%)
Total bilirubin, μmol/L	12.3 (4.1–31.5)
Alanine aminotransferase, U/L	35 (9–64)
Aspartate aminotransferase, U/L	49 (13–72)
Serum albumin, g/L	38.4 (28.9–49.4)
Prealbumin, mg/L	376 (168–459)
Prothrombin time, s	12.6 (10.4–16.6)
Platelet count, ×10^9^/L	240.6 (78.8–696)
Postoperative complications	
Minor (I/II)	96 (76.8%)
Major (III/IV/V)	29 (23.2%)
Liver cirrhosis	77 (61.6%)
Post-hepatectomy liver failure	13 (10.4%)
Portal vein diameter, cm	1.1 (0.7–1.6)
Duration of operation, min	253 (130–450)
Inflow blood occlusion, yes/no	57/68
Blood loss, ml	400 (100–3000)
Blood transfusion	16 (12.8%)
Portal vein tumor thrombus	44 (35.2%)
Tumor diameter, cm	10 (2–22)
Spleen volume, cm^3^	168.5 (46–454)
FLV, cm^3^	1005 (529–1795)
Tumor volume, cm^3^	501 (30.1–2259)
FLRV, cm^3^	601 (265.8–1141)
Resected liver volume, cm^3^	820 (135.5–2617)
%FLRV	0.62 (0.29–0.93)
%Change in remnant liver volume_POW1_	0.213 (0.01–1.092)

Values shown are median (range) or *n* (%).

### Surgical outcomes and complications

No severe intraoperative events occurred. Nearly one quarter of patients (29, 23.2%) experienced major postoperative complications, while three quarters (96, 76.8%) experienced minor complications. Complications included liver failure, pleural effusion, bile leakage, wound infection, renal failure, wound dehiscence, postoperative bleeding, pulmonary infection, portal vein thrombosis, and stress ulcer. Liver failure occurred in 13 patients (10.4%), one of whom died on postoperative day 90 as a result.

### Liver regeneration based on remnant liver volume

**Figure 2:**
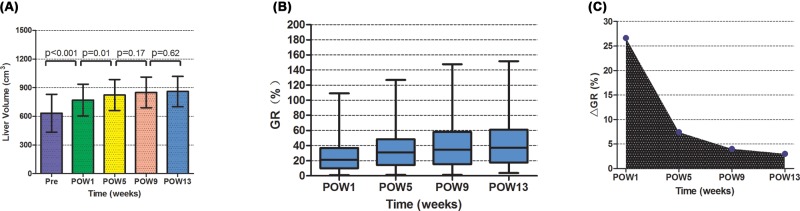
Comparative analysis of liver volume and liver regeneration at different stages postoperatively (**A**) Comparison of preoperative FLRV and postoperative remnant liver volume. (**B**) Box plot showing median and range of liver regeneration growth ratios at different times postoperatively. (**C**) Net growth ratio across all patients at different times postoperatively.

In the entire study population, preoperative FLRV was 633 ± 198 cm^3^, and postoperative remnant liver volume was 771 ± 167 cm^3^ at POW 1, 823 ± 162 cm^3^ at week 5, 851 ± 161 cm^3^ at week 9, and 861 ± 158 cm^3^ at week 13 (Figure 2A). Remnant liver volume at POW 1 was significantly larger than preoperative FLRV, and remnant liver volume was significantly larger at POW 5 than at week 1. However, liver volume did not increase significantly between POWs 9 and 13.

**Figure 3:**
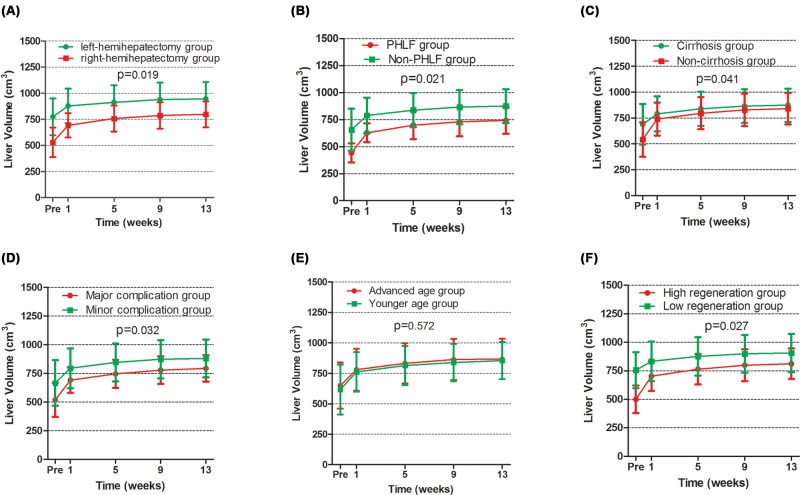
Subgroup analysis of liver volume at different stages postoperative Comparison of liver volume between (**A**) patients undergoing left or right hemihepatectomy, (**B**) patients who experienced PHLF or not, (**C**) patients with or without liver cirrhosis, (**D**) patients experiencing major or minor complications, (**E**) older and younger patients, and (**F**) patients experiencing low or high extents of liver regeneration. Pre, preoperative.

Next, we examined liver regeneration in various binary subgroups stratified according to type of surgery, postoperative liver failure, cirrhosis, complications, age, and extent of regeneration. Remnant liver volume in the right-hemihepatectomy group, liver failure subgroup, non-cirrhosis subgroup, major complications subgroup, or high regeneration subgroup were significantly smaller than that in the corresponding subgroup at all time points (all *P*<0.05; Figure 3). However, remnant liver volume among older patients was similar with that in the younger patients (*P*>0.05; Figure 3E).

### Liver regeneration based on growth ratio

In the entire study population, median growth ratio was 21.3% (range, 1.0–109.2%) at POW 1, 30.9% (range, 1.41–126.9%) at week 5, 34.6% (range, 1.39–147.7%) at week 9, and 37.1% (range, 3.8–151.8%) at week 13 (Figure 2B). Net growth ratios at these time points were 26.6 ± 21.3, 7.4 ± 7.6, 3.6 ± 4.1, and 1.3 ± 2.7% (Figure 2C).

As with remnant liver volume, we next examined growth ratios in various binary subgroups stratified according to type of surgery, postoperative liver failure, cirrhosis, complications, age, and extent of regeneration. Results indicate that both the growth ratio and net growth ratio were higher in the right hemihepatectomy subgroup, patients who experienced liver failure, patients without cirrhosis, patients with major complications, and patients experiencing a high degree of regeneration than the corresponding subgroups (Supplementary Figures S2 and S3, all *P*<0.05). However, older and younger patients showed similar growth ratios and net growth ratios at each time point (Supplementary Figures S2E and S3E, all *P*>0.05).

**Figure 4:**
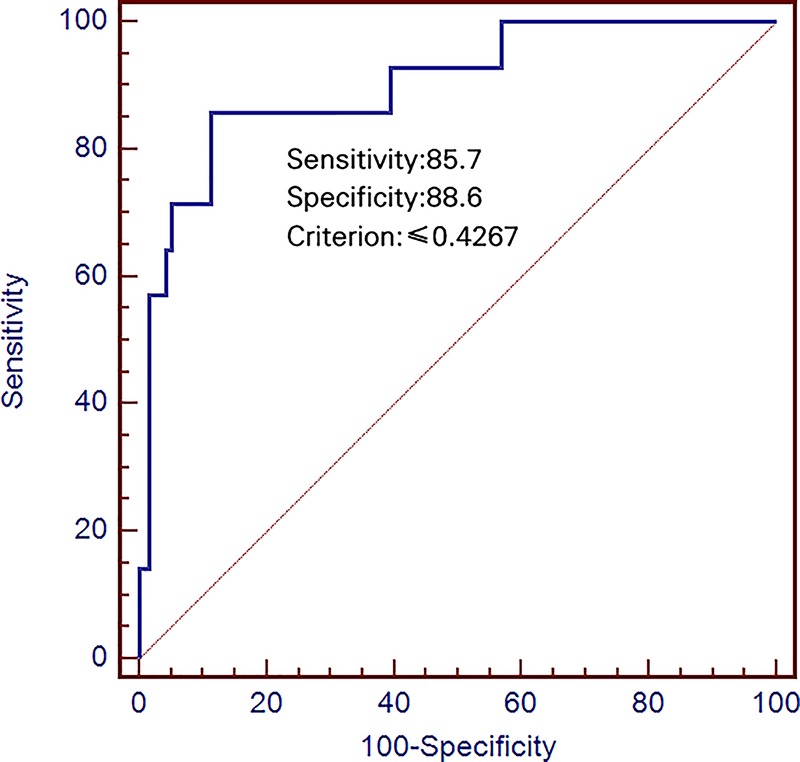
Receiver operating characteristic curve of %FLRV to predict PHLF after hemihepatectomy %FLRV cut-off of 42.7% provided an AUC of 0.900 (95%CI 0.835–0.946, *P*<0.0001).

Analysis of ROC curves indicated that the area under the curve (AUC) was 0.900 for a cut-off %FLRV of 42.7%, which was associated with a sensitivity of 85.7% and specificity of 88.6% (Figure 4).

### Factors associated with high degree of liver regeneration

Numerous parameters related to patients’ pre- and intraoperative status, hepatitis B virus DNA load and treatment history, liver volume, and postoperative complications were examined for possible correlation with high degree of liver regeneration. This univariate analysis identified the following factors associated with high regeneration: postoperative complications (*P*=0.01), inflow blood occlusion time (*P*<0.001), FLRV < 601 cm^3^ (*P*<0.001), %FLRV (*P*<0.001), total liver volume > 1005 cm^3^ (*P*=0.012), tumor volume > 501 cm^3^ (*P*=0.017), and liver cirrhosis (*P*<0.001). Multivariate analysis identified the following factors to be associated with high regeneration (Supplementary Table S1): FLRV < 601 cm^3^ (OR 0.230, 95%CI 0.074–0.717, *P*=0.011), %FLRV (OR 0.271, 95%CI 0.077–0.960, *P*=0.043), and liver cirrhosis (OR 7.740, 95%CI 2.748–21.798, *P*<0.001).

**Figure 5:**
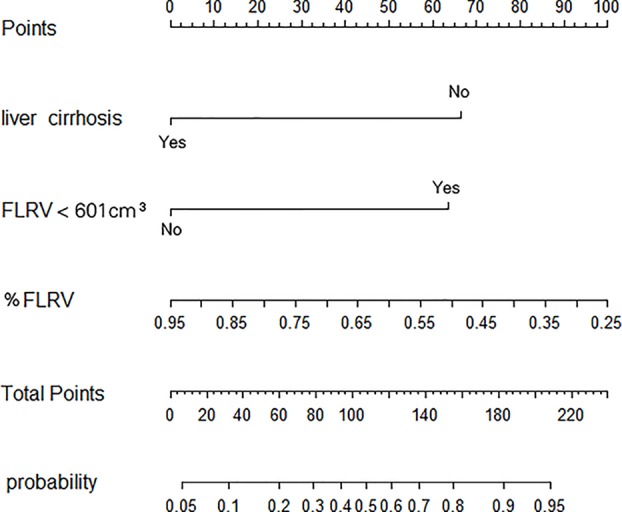
Nomogram to predict the probability of a high degree of liver regeneration after hemihepatectomy Patient’s values are plotted along each axis, and a line is drawn upward to determine the number of points assigned to the values of the variables. The sum of points along the total points axis indicates the probability of high extent of postoperative liver regeneration.

**Figure 6:**
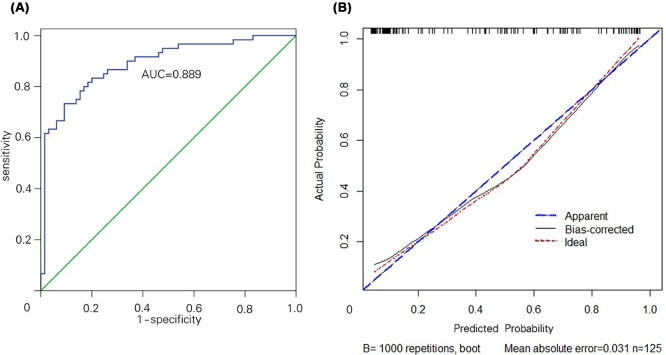
Internal validation of the nomogram to predict the probability of high liver regeneration in hemihepatectomy patients (**A**) Discrimination analysis showed an AUC of 0.889 (95%CI 0.831–0.948). (**B**) Predictive accuracy was assessed using internal validation.

A nomogram model was constructed on the basis of these regeneration predictor (Figure 5), and the model showed an AUC of 0.889 (95%CI 0.831–0.948, *P*<0.001; Figure 6A). Analysis against the internal validation dataset suggested a strong ability to predict high degree of liver regeneration (Figure 6B).

### Influence of liver regeneration on recovery of liver function

**Figure 7:**
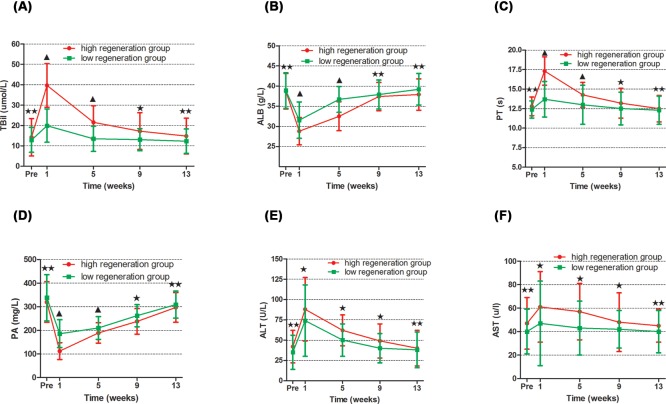
Comparison of liver function indices between patients who experienced high or low extents of post-hepatectomy liver regeneration (**A**) Tbil; (**B**) ALB; (**C**) PT; (**D**) PA; (**E**) ALT; (**F**) AST. ^▲^*P*<0.05, ^★^*P*>0.05, ^★★^*P*>0.10. ALB, serum albumin; ALT, alanine aminotransferase; AST, aspartate aminotransferase; PA, prealbumin; Pre, preoperative; PT, prothrombin time; TBiL, total bilirubin.

The two subgroups showing low or high extent of regeneration showed similar liver function preoperatively and at POWs 9 and 13 (all *P*>0.05; Figure 7). However, at weeks 1 and 5, the liver function biological parameters (TBil, PT) were significantly higher and ALB, PA were significantly lower in the high regeneration group than low regeneration group (all *P*<0.05; Figure 7). AST and ALT were not significantly different between these two groups (all *P*>0.05; Figure 7).

## Discussion

Liver regeneration following parenchymal damage, hepatectomy, or injury reflects compensatory hyperplasia in which the residual liver expands to meet metabolic need [19,20]. Regeneration can be studied accurately using imaging technologies [21–23], and studies of liver regeneration following transplantation into HCC patients or following hepatectomy [24–27] suggest that regeneration proceeds rapidly during the first 2 weeks, then slows, and then gently increases [26,28–31], leading to liver expansion of 28–64% by up to 6 months after hepatectomy [26,28,32]. This range reflects heterogeneity in the types of hepatectomy included in the studies. The present study included only patients who underwent hemihepatectomy, for which pre- and postoperative liver volume can be measured objectively using imaging software, without influence from the doctor performing the surgery. Our study found that right hemihepatectomy, PHLF, major complications, absence of liver cirrhosis and low degree of postoperative liver regeneration were associated with much lower FLRV and remnant liver volume (Figure 3). The volume of the right was generally larger than the left lobe. So, patients who underwent right hemihepatectomy were taken for granted less FLRV and remnant liver volume. With the improvement of perioperative management and hepatic surgery technology, the occurrence of postoperative complication and PHLF have a close relationship with the less remnant liver volume [33]. Our research results were consistent with those published results. Our study also found that patient age did not seem to influence FLRV or remnant liver volume (Figure 3E). In one study [34], which included 41 HCC patients who underwent major hepatectomy, found that liver regeneration was not affected by age. This result was in accordance with ours.

In our study, remnant liver showed 21.3% regeneration by POW 1 and 30.9% by week 5, but the regeneration growth ratio was not significant at longer time points, and the net growth ratio declined dramatically after week 5. Overall, our results suggest rapid regeneration within 1 week after hepatectomy, followed by slower growth out to week 5, and then stable liver volume between weeks 9 and 13. Subgroup analysis showed that growth ratio depended on whether the hemihepatectomy was right or left, whether the patient experienced liver failure or not, and whether the patient experienced major or minor complications. The growth ratio did not, however, depend on patient age (Supplementary Figure S2). Our results are consistent with previous work [29,30,32,35,36] showing that functional liver regeneration occurs early and rapidly in older and younger patients alike. At the same time, one study [37] found age to correlate inversely with early liver regeneration in patients undergoing major hepatectomy, and those authors concluded that massive blood loss should be avoided to ensure early liver regeneration in older patients [37].

We found much higher FLRV and remnant liver volume as well as lower growth ratio in patients with liver cirrhosis than in patients without it. Nevertheless, these results do not allow clear conclusions about a potential influence of cirrhosis on liver regeneration since the resected liver tissue served as the basis for pathology–based diagnosis of cirrhosis, yet regeneration was induced by the remnant liver tissue, and cirrhosis severity can differ even within different sections of the same liver. Further work is needed to explore this potential association. A mouse study suggests no influence of cirrhosis on liver regeneration [38], but more clinical studies are needed.

What triggers liver regeneration after hepatectomy is poorly understood, in part because previous studies cannot take into account all the factors potentially involved. Candidate triggers include FLRV before portal vein embolization [15,39], chronic liver disease [40,41], diabetes [42], major portal hypertension [43], chemotherapy [44,45], certain hematological parameters [46,47], and a rapid increase in portal vein blood flow to the remnant liver tissue [48,49]. In our study, multivariate analysis identified FLRV, %FLRV, and liver cirrhosis as predictors of high degree of liver regeneration at POW 1. A previous study [15] similarly identified %FLRV as independently correlated with liver regeneration after hepatectomy. This factor seems a reasonable predictor of regeneration since if it is insufficient, the patient may experience liver failure rather than successful liver regeneration. In our patient population, a %FLRV cut–off of 42.7% was associated with liver failure (Figure 4).

Our analysis of potential predictors of high liver regeneration allowed us to construct a nomogram that discriminated well between patients who experienced low or high degrees of regeneration and showed an AUC of 0.889 (95%CI 0.831–0.948, *P*<0.001; Figure 6A). The nomogram performed well at predicting high regeneration among the internal validation dataset (Figure 6B). It may prove useful for wider clinical use, although it should first be carefully tested, refined, and validated in different clinical settings and different patient backgrounds.

While previous studies [24,50] have shown that post-hepatectomy liver regeneration strongly influences recovery of liver function, which can take from 2 weeks to several months depending on the liver background and degree of injury to liver parenchyma [31,51–53], we found that patients who experienced high degree of liver regeneration recovered liver function more slowly than those who experienced low degree of regeneration (Figure 7). This phenomenon may be related to the smaller residual liver volume (Figure 3) and poor function in those patients in high regeneration group. In theory, it will meet the patients’ physiological needs while the liver volume increases to a certain degree. However, At the stage of liver volume increasing post-hepatectomy, the hyperplasia liver cells cannot completely provide normal liver cell function. So, these findings likely reflect the inverse relationship between FLRV and postoperative liver regeneration, and they illustrate that liver volume does not necessarily represent liver function during regeneration [54,55]. This is consistent with several studies showing that liver cell mitosis does not always correlate with hepatic metabolism or detoxification [20,56–58]. In addition, the two subgroups showed similar recovery of liver function indicators by POWs 9 and 13. At this stage, it is enough to maintain the liver function because of the recovery for the hyperplasia liver cell.

The results of the present study should be interpreted with caution in light of several limitations. The sample was small and came from a single hospital, which meant that we could validate our predictive modeling internally but not externally using patients from another institution or another time period. In addition, we did not take into account several clinical factors that might affect liver regeneration, including steatosis, portal vein flow, spleen size, liver tissue edema, inflammation, and hepatic B virus activity, and so on.

Despite these limitations, our results provide evidence that FLRV <601 cm^3^, %FLRV and liver cirrhosis can strongly influence liver regeneration after hemihepatectomy. Taking into account only these clinical factors allowed us to build a simple nomogram capable of accurately predicting probability of high degree of liver regeneration. Our results also suggest 42.7% as the minimum %FLRV needed to avoid elevated risk of PHLF. Our results, together with previous studies, indicate that liver regeneration proceeds fastest during the week following hemihepatectomy, then it slows down during the subsequent month and stabilizes in the longer term. Regeneration of liver volume does not directly correlate with recovery of liver function, and in fact these two outcomes may correlate inversely.

## Supporting information

**Supplementary Figure S1:** 

**Supplementary Figure S2:** 

**Supplementary Figure S3:** 

**Supplementary Table S1:** Uni- and multivariate logistic regression to identify predictors of high extent of liver regeneration after hemihepatectomy.

## Abbreviations

AUC: area under the curve
CT: computed tomography
FLRV: future liver remnant volume
FLV: functional liver volume
HBsAg: hepatitis B surface antigen
HCC: hepatocellular carcinoma
PHLF: post-hepatectomy liver failure
POW: postoperative week
ROC: receiver operating characteristic
TACE: transarterial chemoembolization
