# Supplementary material for: Evaluation of liver regeneration and post-hepatectomy liver failure after hemihepatectomy in patients with hepatocellular carcinoma
Source: Biosci Rep. 2019 Aug 23;39(8):BSR20190088. doi: 10.1042/BSR20190088 (PMC6706596; doi:10.1042/BSR20190088)
Supplement: Supplementary file 1 [file bsr20190088_Supp1.pdf]

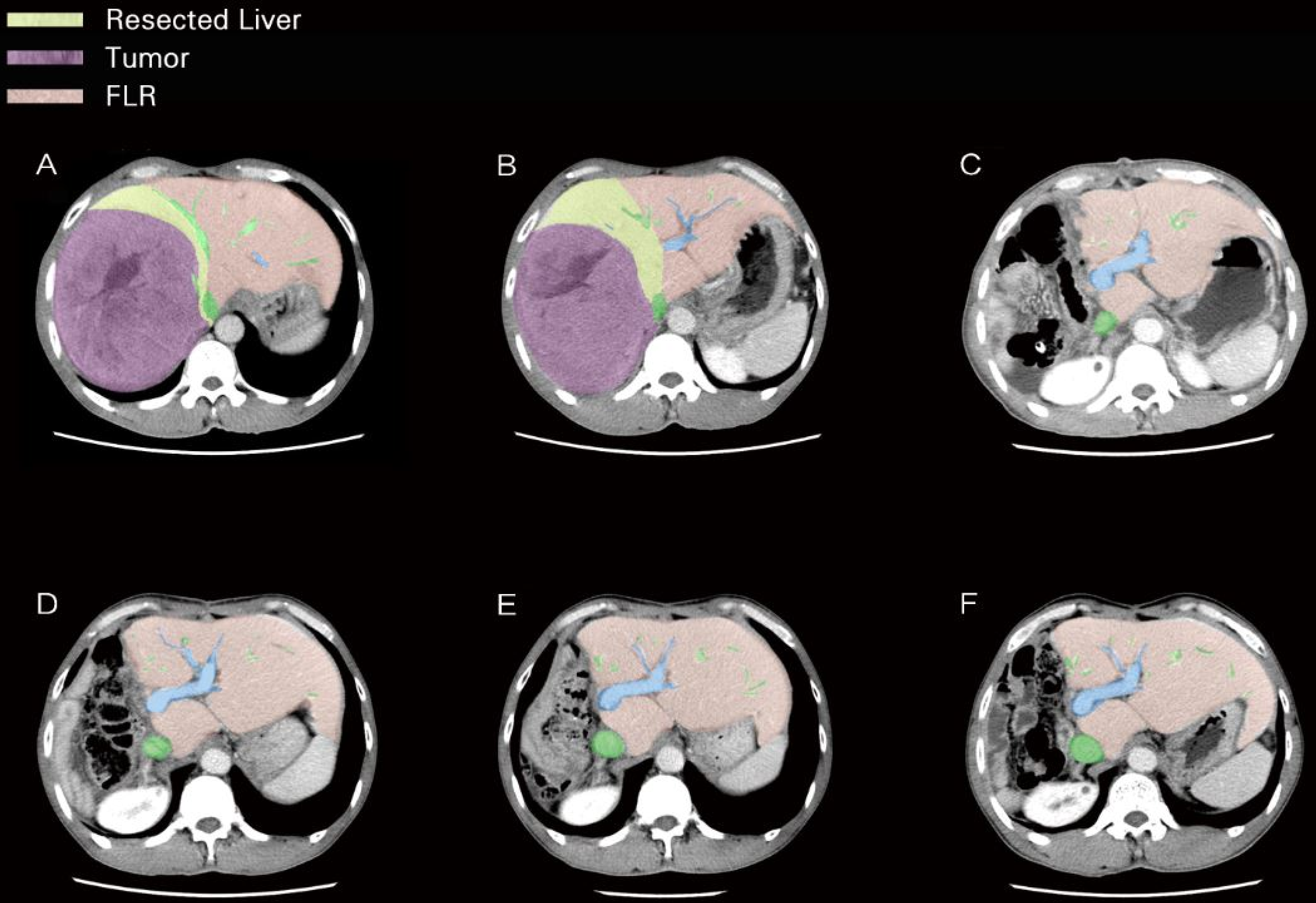
Resected Liver
Tumor
FLR
A
B
C
D
E
F

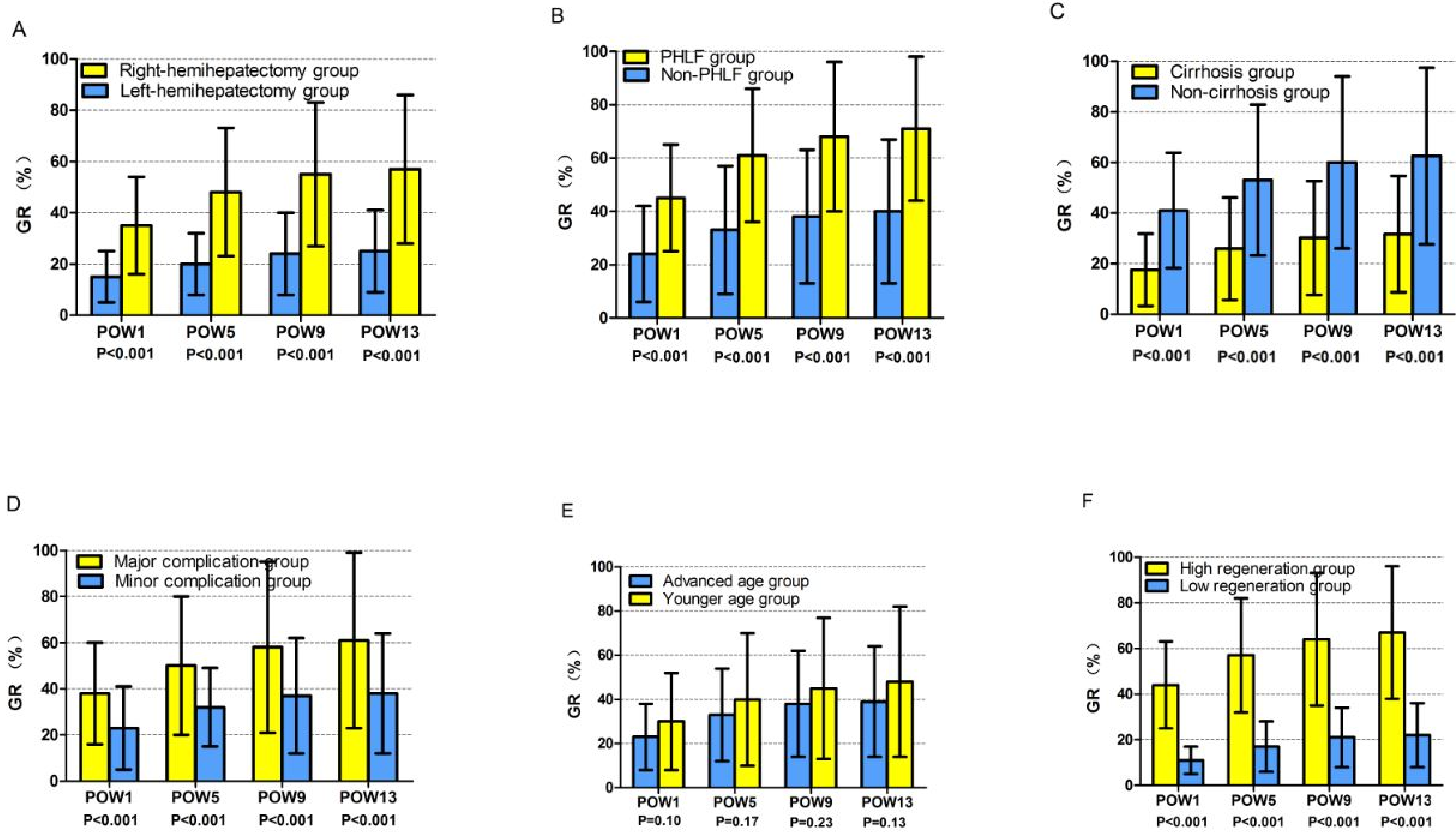
A
Right-hemihepatectomy group
Left-hemihepatectomy group
GR (%)
POW1 P<0.001
POW5 P<0.001
POW9 P<0.001
POW13 P<0.001
B
PHLF group
Non-PHLF group
GR (%)
POW1 P<0.001
POW5 P<0.001
POW9 P<0.001
POW13 P<0.001
C
Cirrhosis group
Non-cirrhosis group
GR (%)
POW1 P<0.001
POW5 P<0.001
POW9 P<0.001
POW13 P<0.001
D
Major complication group
Minor complication group
GR (%)
POW1 P<0.001
POW5 P<0.001
POW9 P<0.001
POW13 P<0.001
E
Advanced age group
Younger age group
GR (%)
POW1 P=0.10
POW5 P=0.17
POW9 P=0.23
POW13 P=0.13
F
High regeneration group
Low regeneration group
GR (%)
POW1 P<0.001
POW5 P<0.001
POW9 P<0.001
POW13 P<0.001

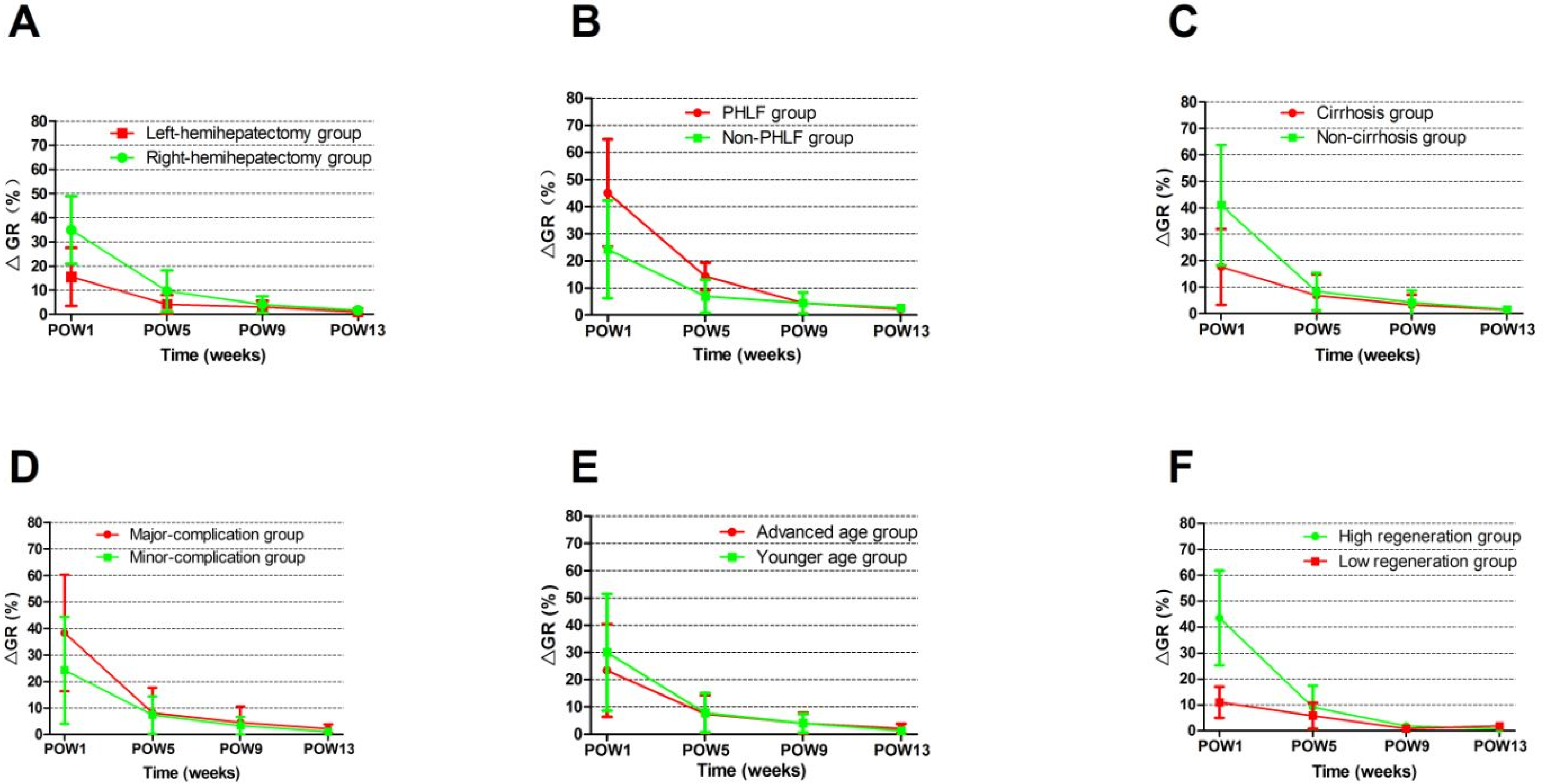

A
Left-hemihepatectomy group
Right-hemihepatectomy group
△GR（%）
Time (weeks)
POW1
POW5
POW9
POW13
B
PHLF group
Non-PHLF group
△GR（%）
Time (weeks)
C
Cirrhosis group
Non-cirrhosis group
△GR (%)
Time (weeks)
D
Major-complication group
Minor-complication group
△GR (%)
Time (weeks)
E
Advanced age group
Younger age group
△GR (%)
Time (weeks)
F
High regeneration group
Low regeneration group
△GR (%)
Time (weeks)

Table S1. Uni- and multivariate logistic regression to identify predictors of high extent of liver regeneration after hemihepatectomy.

Variable (n=125)	Univariate *P*	Multivariate OR (95%CI)	*P*
Gender (Male)	0.792		
Age* (> 47 yr)	0.647		
Body mass index* (>21.5 kg/m^2)	0.282		
Body surface area* (>1.61 m^2)	0.132		
Diabetes	0.375		
Positive for Hepatitis B Virus DNA	0.551		
Antiviral therapy	0.200		
Alpha fetoprotein (≥200/<200ug/ml)	0.829		
Total bilirubin * (>12.3 μmol/L)	0.183		
Serum albumin* (>38.4 g/L)	0.786		
Alanine aminotransferase * (>40 U/L)	0.100		
Aspartate aminotransferase * (>40 U/L)	0.179		
Prothrombin time * (>12.6 sec)	0.932		
Platelet count* (>240.6 ×10^9/L)	0.920		
MELD score (>24)	0.343		
Portal vein tumor thrombus	0.242		
Duration of operation* (>253 min)	0.871		
Blood loss* (>400 ml)	0.343		
Blood transfusion	0.479		
Spleen volume* (>168.5 cm^3)	0.629		
Resected liver volume* (>820 cm^3)	0.382		
Liver cirrhosis	0.001	7.740 (2.748-21.798)	<0.001
Inflow blood occlusion (yes)	0.004	0.519 (0.188-1.439)	0.208
Postoperative complications	0.012	1.588 (0.472-5.340)	0.455
FLV* (>1005 cm^3)	0.008	0.334 (0.101-1.106)	0.073
Tumor volume* (>501 cm^3)	0.017	1.234 (0.439-3.466)	0.690

FLRV* (<601 cm^3)	0.004	0.230 (0.074-0.717)	0.011
%FLRV	0.001	0.271 (0.077-0.960)	0.043

* Median values in the study population were used as cut-off values.

FLRV: Future Liver Remnant Volume; FLV: Functional Liver Volume
